# *N*-Phenylpyridine-2-carb­amide

**DOI:** 10.1107/S1600536808028274

**Published:** 2008-09-13

**Authors:** Yu-Guo Zhuang, Hua-Jiang Jiang, Zhi Hong, Fang-Li Qiu

**Affiliations:** a School of Pharmaceutical and Chemical Engineering, Taizhou University, Linhai 317000, People’s Republic of China

## Abstract

In the title compound, C_12_H_10_N_2_O, the dihedral angle between the pyridine ring system and the phenyl ring is 1.8 (1)°. There is an intra­molecular N—H⋯N hydrogen bond between the pyridine N atom and the amide NH function.

## Related literature

For general background, see: Sousa & Filgueiras (1990 ▶); Gomes *et al.* (2007 ▶); Morsali *et al.* (2003 ▶); Jacob & Mukherjee (2006 ▶); Marumoto *et al.* (1981 ▶); Piatnitski & Kiselyov (2004 ▶). For related structures, see: Qi *et al.* (2003 ▶); Zhang *et al.* (2006 ▶); Yin *et al.* (2007 ▶). For the synthesis, see: Chan *et al.* (2004 ▶).
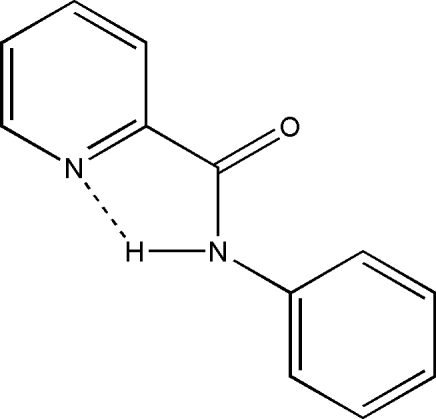


## Experimental

### 

#### Crystal data

C_12_H_10_N_2_O
*M* *_r_* = 198.22
Monoclinic, 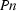

*a* = 5.7469 (2) Å
*b* = 6.2382 (2) Å
*c* = 14.0158 (3) Å
β = 94.752 (2)°
*V* = 500.74 (3) Å^3^
*Z* = 2
Mo *K*α radiation
μ = 0.09 mm^−1^
*T* = 296 (2) K
0.15 × 0.14 × 0.09 mm

#### Data collection

Bruker APEXII CCD diffractometer
Absorption correction: multi-scan (*SADABS*; Bruker, 2004 ▶) *T*_min_ = 0.968, *T*_max_ = 0.992
5000 measured reflections
1162 independent reflections
1036 reflections with *I* > 2σ(*I*)
*R*_int_ = 0.017

#### Refinement

*R*[*F* ^2^ > 2σ(*F* ^2^)] = 0.030
*wR*(*F* ^2^) = 0.075
*S* = 1.00
1162 reflections
137 parameters
H-atom parameters constrained
Δρ_max_ = 0.11 e Å^−3^
Δρ_min_ = −0.12 e Å^−3^

### 

Data collection: *APEX2* (Bruker, 2004 ▶); cell refinement: *SAINT-Plus* (Bruker, 2004 ▶); data reduction: *SAINT-Plus*; program(s) used to solve structure: *SHELXL97* (Sheldrick, 2008 ▶); program(s) used to refine structure: *SHELXL97* (Sheldrick, 2008 ▶); molecular graphics: *ORTEP-3 for Windows* (Farrugia, 1997 ▶); software used to prepare material for publication: *WinGX* (Farrugia,1999 ▶).

## Supplementary Material

Crystal structure: contains datablocks I, global. DOI: 10.1107/S1600536808028274/im2083sup1.cif

Structure factors: contains datablocks I. DOI: 10.1107/S1600536808028274/im2083Isup2.hkl

Additional supplementary materials:  crystallographic information; 3D view; checkCIF report

## Figures and Tables

**Table 1:** Hydrogen-bond geometry (Å, °)

*D*—H⋯*A*	*D*—H	H⋯*A*	*D*⋯*A*	*D*—H⋯*A*
N1—H101⋯N2	0.86	2.28	2.697 (2)	110

**Fig. 1.**
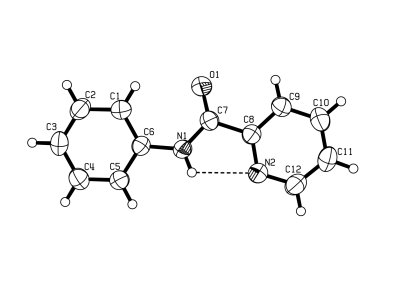
The molecular structure of the title compound, shown with 50% probability displacement ellipsoids.

## supplementary crystallographic information

### Comment

Pyridine-containing amides continue to attract considerable interest as ligands for metals (Sousa & Filgueiras, 1990; Gomes *et al.*, 2007; Jacob & Mukherjee, 2006), building blocks in organic synthesis (Marumoto *et al.*, 1981) and physiologically active compounds (Piatnitski & Kiselyov, 2004). As part of our studies on the synthesis and characterization of these compounds, we report here the crystal structure of the title compound.

The C7—O1 [1.223 (3) Å], N1—C7 [1.344 (3) Å] and N1—C6 [1.410 (2) Å] bond lengths indicate extensive electron delocalization in the amide linkage. The pyridyl and phenyl rings of the title compound are almost coplanar, forming a dihedral angle of 1.8 (1)°. In the crystal structure, there is an intramolecular hydrogen bond (N1—H101···N2) and no intermolecular hydrogen bonds are observed (Table 1).

The reported monoclinic space-group is in a non-standard setting (*Pn*, #7). There is a strong feature (*h* + l = 2*n*) in *hkl* data. Setting up the space group as *Pc* results in a β angle of 23° or 157°, respectively. Obviously such an unit-cell division is inappropriate. Therefore, the non-standard setting *Pn* was chosen.

### Experimental

The title compound was synthesized from pyridine-2-carboxylic acid and aniline according to the procedure of Chan *et al.* (2004). The crystal used for data collection was obtained by slow evaporation from a saturated ethanol/water solution at room temperature.

### Refinement

All H atoms were placed in geometrically idealized positions and constrained to ride on their parent atoms, with N—H = 0.86 Å, C—H = 0.93 Å, and with *U*_iso_(H) = 1.2*U*_eq_(parent atom).

### Crystal data

**Table:**

C_12_H_10_N_2_O	*F*(000) = 208
*M**_r_* = 198.22	*D*_x_ = 1.315 Mg m^−^^3^
Monoclinic, *P**n*	Mo *K*α radiation, λ = 0.71073 Å
Hall symbol: P -2yac	Cell parameters from 2226 reflections
*a* = 5.7469 (2) Å	θ = 2.9–25.1°
*b* = 6.2382 (2) Å	µ = 0.09 mm^−^^1^
*c* = 14.0158 (3) Å	*T* = 296 K
β = 94.752 (2)°	Block, colourless
*V* = 500.74 (3) Å^3^	0.15 × 0.14 × 0.09 mm
*Z* = 2	

### Data collection

**Table:**

Bruker APEXII CCD diffractometer	1162 independent reflections
Radiation source: fine-focus sealed tube	1036 reflections with *I* > 2σ(*I*)
graphite	*R*_int_ = 0.017
Detector resolution: 10 pixels mm^-1^	θ_max_ = 27.7°, θ_min_ = 2.9°
ω scans	*h* = −7→7
Absorption correction: multi-scan (SADABS; Bruker, 2004)	*k* = −8→5
*T*_min_ = 0.968, *T*_max_ = 0.992	*l* = −18→18
5000 measured reflections	

### Refinement

**Table:**

Refinement on *F*^2^	Primary atom site location: structure-invariant direct methods
Least-squares matrix: full	Secondary atom site location: difference Fourier map
*R*[*F*^2^ > 2σ(*F*^2^)] = 0.030	Hydrogen site location: inferred from neighbouring sites
*wR*(*F*^2^) = 0.075	H-atom parameters constrained
*S* = 1.00	*w* = 1/[σ^2^(*F*_o_^2^) + (0.0365*P*)^2^ + 0.0548*P*] where *P* = (*F*_o_^2^ + 2*F*_c_^2^)/3
1162 reflections	(Δ/σ)_max_ < 0.001
137 parameters	Δρ_max_ = 0.11 e Å^−^^3^
0 restraints	Δρ_min_ = −0.12 e Å^−^^3^

### Special details

**Table:**

Geometry. All e.s.d.'s (except the e.s.d. in the dihedral angle between two l.s. planes) are estimated using the full covariance matrix. The cell e.s.d.'s are taken into account individually in the estimation of e.s.d.'s in distances, angles and torsion angles; correlations between e.s.d.'s in cell parameters are only used when they are defined by crystal symmetry. An approximate (isotropic) treatment of cell e.s.d.'s is used for estimating e.s.d.'s involving l.s. planes.
Refinement. Refinement of *F*^2^ against ALL reflections. The weighted *R*-factor *wR* and goodness of fit *S* are based on *F*^2^, conventional *R*-factors *R* are based on *F*, with *F* set to zero for negative *F*^2^. The threshold expression of *F*^2^ > σ(*F*^2^) is used only for calculating *R*-factors(gt) *etc*. and is not relevant to the choice of reflections for refinement. *R*-factors based on *F*^2^ are statistically about twice as large as those based on *F*, and *R*- factors based on ALL data will be even larger.

### Fractional atomic coordinates and isotropic or equivalent isotropic displacement parameters (Å^2^)

**Table:**

	*x*	*y*	*z*	*U*_iso_*/*U*_eq_	
N1	−0.0173 (3)	0.2086 (3)	0.59409 (11)	0.0452 (4)	
H101	−0.1420	0.2510	0.6183	0.054*	
C8	0.1414 (3)	0.5113 (3)	0.68241 (13)	0.0435 (4)	
C6	−0.0401 (3)	0.0235 (3)	0.53631 (12)	0.0412 (4)	
C1	0.1246 (4)	−0.0375 (4)	0.47407 (15)	0.0509 (5)	
H1	0.2570	0.0459	0.4685	0.061*	
C7	0.1751 (4)	0.3270 (3)	0.61595 (15)	0.0475 (5)	
C2	0.0901 (4)	−0.2230 (4)	0.42049 (16)	0.0569 (5)	
H2	0.1999	−0.2635	0.3787	0.068*	
N2	−0.0537 (3)	0.5139 (3)	0.72828 (12)	0.0526 (4)	
C5	−0.2373 (4)	−0.1016 (3)	0.54213 (15)	0.0476 (5)	
H5	−0.3507	−0.0595	0.5819	0.057*	
O1	0.3656 (3)	0.2937 (3)	0.58575 (15)	0.0753 (6)	
C10	0.2767 (5)	0.8384 (3)	0.75234 (17)	0.0575 (5)	
H10	0.3863	0.9481	0.7597	0.069*	
C11	0.0794 (4)	0.8441 (4)	0.80026 (16)	0.0612 (6)	
H11	0.0523	0.9575	0.8410	0.073*	
C3	−0.1044 (4)	−0.3483 (4)	0.42826 (16)	0.0553 (5)	
H3	−0.1255	−0.4731	0.3923	0.066*	
C12	−0.0787 (4)	0.6788 (4)	0.78711 (17)	0.0629 (6)	
H12	−0.2107	0.6823	0.8213	0.076*	
C9	0.3106 (4)	0.6677 (3)	0.69302 (15)	0.0516 (5)	
H9	0.4451	0.6581	0.6607	0.062*	
C4	−0.2676 (4)	−0.2879 (4)	0.48961 (17)	0.0553 (5)	
H4	−0.3986	−0.3728	0.4956	0.066*	

### Atomic displacement parameters (Å^2^)

**Table:**

	*U*^11^	*U*^22^	*U*^33^	*U*^12^	*U*^13^	*U*^23^
N1	0.0458 (9)	0.0397 (9)	0.0515 (9)	−0.0007 (7)	0.0121 (7)	−0.0031 (7)
C8	0.0478 (10)	0.0408 (10)	0.0422 (10)	0.0013 (8)	0.0051 (8)	0.0021 (8)
C6	0.0458 (10)	0.0369 (10)	0.0408 (10)	0.0030 (8)	0.0042 (8)	0.0021 (8)
C1	0.0504 (11)	0.0521 (12)	0.0517 (11)	−0.0041 (10)	0.0120 (9)	−0.0028 (10)
C7	0.0497 (11)	0.0398 (11)	0.0539 (12)	−0.0030 (9)	0.0092 (9)	−0.0007 (9)
C2	0.0594 (13)	0.0609 (14)	0.0515 (12)	0.0060 (11)	0.0117 (9)	−0.0103 (10)
N2	0.0477 (9)	0.0540 (10)	0.0566 (10)	−0.0015 (8)	0.0067 (8)	−0.0096 (8)
C5	0.0464 (10)	0.0458 (11)	0.0515 (10)	−0.0004 (9)	0.0088 (8)	−0.0024 (9)
O1	0.0531 (9)	0.0669 (11)	0.1095 (15)	−0.0111 (9)	0.0283 (9)	−0.0330 (10)
C10	0.0699 (14)	0.0471 (11)	0.0550 (11)	−0.0102 (12)	0.0014 (10)	−0.0007 (11)
C11	0.0714 (16)	0.0553 (14)	0.0559 (13)	0.0065 (12)	−0.0002 (11)	−0.0156 (11)
C3	0.0652 (13)	0.0459 (11)	0.0537 (11)	0.0014 (11)	−0.0023 (10)	−0.0119 (10)
C12	0.0561 (13)	0.0705 (15)	0.0635 (14)	0.0047 (11)	0.0122 (11)	−0.0168 (12)
C9	0.0559 (11)	0.0486 (12)	0.0513 (11)	−0.0086 (10)	0.0116 (9)	−0.0018 (9)
C4	0.0550 (12)	0.0506 (13)	0.0598 (12)	−0.0070 (10)	0.0027 (10)	−0.0027 (11)

### Geometric parameters (Å, °)

**Table:**

N1—C7	1.344 (3)	N2—C12	1.333 (3)
N1—C6	1.410 (2)	C5—C4	1.379 (3)
N1—H101	0.8600	C5—H5	0.9300
C8—N2	1.338 (2)	C10—C11	1.365 (4)
C8—C9	1.377 (3)	C10—C9	1.375 (3)
C8—C7	1.502 (3)	C10—H10	0.9300
C6—C5	1.384 (3)	C11—C12	1.377 (3)
C6—C1	1.392 (3)	C11—H11	0.9300
C1—C2	1.385 (3)	C3—C4	1.377 (3)
C1—H1	0.9300	C3—H3	0.9300
C7—O1	1.223 (3)	C12—H12	0.9300
C2—C3	1.376 (4)	C9—H9	0.9300
C2—H2	0.9300	C4—H4	0.9300
			
C7—N1—C6	128.07 (17)	C4—C5—H5	119.6
C7—N1—H101	116.0	C6—C5—H5	119.6
C6—N1—H101	116.0	C11—C10—C9	118.9 (2)
N2—C8—C9	123.48 (19)	C11—C10—H10	120.6
N2—C8—C7	117.62 (17)	C9—C10—H10	120.6
C9—C8—C7	118.89 (18)	C10—C11—C12	118.7 (2)
C5—C6—C1	119.06 (17)	C10—C11—H11	120.7
C5—C6—N1	117.74 (17)	C12—C11—H11	120.7
C1—C6—N1	123.19 (17)	C2—C3—C4	119.6 (2)
C2—C1—C6	119.57 (19)	C2—C3—H3	120.2
C2—C1—H1	120.2	C4—C3—H3	120.2
C6—C1—H1	120.2	N2—C12—C11	123.8 (2)
O1—C7—N1	124.81 (19)	N2—C12—H12	118.1
O1—C7—C8	120.61 (18)	C11—C12—H12	118.1
N1—C7—C8	114.58 (18)	C10—C9—C8	118.7 (2)
C3—C2—C1	120.8 (2)	C10—C9—H9	120.6
C3—C2—H2	119.6	C8—C9—H9	120.6
C1—C2—H2	119.6	C3—C4—C5	120.1 (2)
C12—N2—C8	116.38 (18)	C3—C4—H4	119.9
C4—C5—C6	120.77 (19)	C5—C4—H4	119.9

### Hydrogen-bond geometry (Å, °)

**Table:**

*D*—H···*A*	*D*—H	H···*A*	*D*···*A*	*D*—H···*A*
N1—H101···N2	0.86	2.28	2.697 (2)	110.
